# GENETIC INFLUENCES ON LUNG FUNCTION CONTRIBUTE TO SUBSEQUENT AGE CHANGES IN MOTOR FUNCTION

**DOI:** 10.1093/geroni/igz038.2389

**Published:** 2019-11-08

**Authors:** Deborah G Finkel, Marie Ernsth-Bravell

**Affiliations:** 1 Indiana University Southeast, New Albany, Indiana, United States; 2 Jönköping University, Jönköping, Sweden

## Abstract

Researchers have striven to determine whether the age changes in physical and cognitive functioning are coincident, or does change in one domain precede functioning in the other. Dual change score models (DCSM) facilitate testing of hypotheses about temporal patterns of aging. Previous investigations in the Swedish Adoption/Twin Study of Aging (SATSA) indicate three directional effects: age changes in processing speed contribute to subsequent age changes in cognition, age changes in lung function contribute to subsequent age changes in processing speed, and age changes in motor function contribute to subsequent age changes in processing speed. In the current analysis we apply DCSM to twin data to examine the nature of the longitudinal relationship between motor functioning and lung function. Three motor functioning factors were created from 20 performance measures: balance, flexibility, and fine motor movement. Peak expiratory flow measured lung function. Participants were 829 adults aged 50-88 at the first of 9 waves of testing (mean = 4.4 waves) covering a 27-year follow-up period (mean = 13.1 years). Model comparisons indicated that genetic influences on decline in lung function contributed to subsequent decline in motor function. Combined with previous results, these results suggest a pathway that may start with age declines in lung function, which then contribute to declines in motor function, which in turn contribute to subsequent declines in processing speed and then cognitive decline. These data indicate that interventions focusing on improving or maintain lung function should have the added effect of maintaining motor and cognitive function.

